# Role of BACE1 in Alzheimer’s synaptic function

**DOI:** 10.1186/s40035-017-0093-5

**Published:** 2017-08-30

**Authors:** Brati Das, Riqiang Yan

**Affiliations:** Department of Neurosciences, Lerner Research Institute, Cleveland Clinic, 9500 Euclid Avenue/NC30, Cleveland, OH 44195 USA

**Keywords:** Amyloid deposition, β-amyloid peptide, BACE1, Secretase, BACE1 substrates, Synaptic functions

## Abstract

Alzheimer’s disease (AD) is the most common age-dependent disease of dementia, and there is currently no cure available. This hallmark pathologies of AD are the presence of amyloid plaques and neurofibrillary tangles. Although the exact etiology of AD remains a mystery, studies over the past 30 have shown that abnormal generation or accumulation of β-amyloid peptides (Aβ) is likely to be a predominant early event in AD pathological development. Aβ is generated from amyloid precursor protein (APP) via proteolytic cleavage by β-site APP cleaving enzyme 1 (BACE1). Chemical inhibition of BACE1 has been shown to reduce Aβ in animal studies and in human trials. While BACE1 inhibitors are currently being tested in clinical trials to treat AD patients, it is highly important to understand whether BACE1 inhibition will significantly impact cognitive functions in AD patients. This review summarizes the recent studies on BACE1 synaptic functions. This knowledge will help to guide the proper use of BACE1 inhibitors in AD therapy.

## Background

Alzheimer’s disease (AD) is an age-dependent chronic neurodegenerative disease that is characterized by the presence of amyloid deposition, neurofibrillary tangles, synaptic dysfunction, and neuronal cell death [1, 2]. The common effector of this neurodegenerative process is the excessive production or accumulation of β-amyloid (Aβ), which has several deleterious effects on synaptic activity [3, 4]. For over 30 years, amyloid precursor protein (APP) has been a main target for investigating the progression of AD. Aβ is generated from APP through proteolysis in a two-step process: β-secretase, known as β-site APP cleaving enzyme 1 (BACE1), initiates the cleavage of APP to release the membrane-anchored C-terminal fragment, and then γ-secretase subsequently cleaves this fragment to excise Aβ in 40–43 amino acid sequences [5]. These sequences form hydrophobic aggregates, which constitute the senile plaques in AD. Risk factors associated with development of AD pathology involve genetic predisposition (familial early-onset forms), allele forms of apolipoprotein E (i.e, ApoE-4 has the strongest impact), age, lifestyle, and converging evidence which suggests that many newly identified mutations are linked to altered APP processing leading to amyloidogenic pathogenesis [6, 7].

**Fig. 1:**
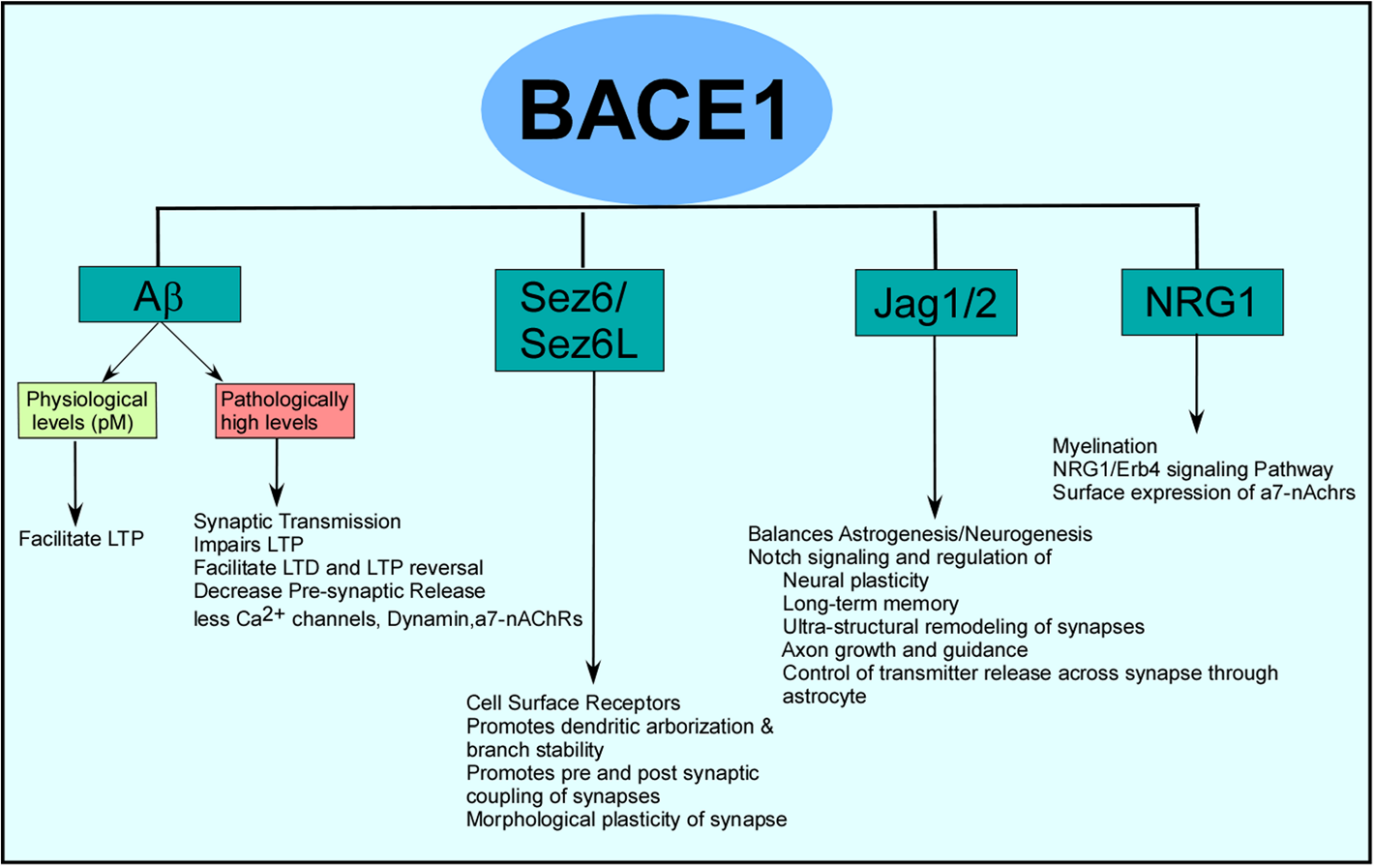
APP and non-APP BACE1 substrates and their effects on synaptic transmisssion

BACE1 has been an important target for therapeutic intervention because of its indispensable role in the generation of Aβ [8–12]. However, BACE1 also functions as a housekeeping enzyme and is involved in the processing of many other proteins that are responsible for proper functioning of neuronal tissue [Fig. 1]. Hence, complete removal of BACE1 enzymatic activity could potentially cause unwanted side effects. The most relevant of these is the effect of BACE1 on synaptic functions, which are related to AD pathology. To this end, this review aims to summarize our knowledge associated with the beneficial and detrimental effects of BACE1 in synaptic functions so that we can have a clearer understanding of the synaptic regulation by BACE1. This understanding will ultimately be beneficial for finding an optimally effective strategy to provide BACE1 drugs to AD patients.

## Pathophysiology of AD and synaptic deficiencies

AD, along with stroke, is the third most common disease affecting the US population, afflicting ~7% of individuals ages 65–74, 53% at ages 75–84, and 40% at ages 85 and older, respectively [13]. Worldwide, it affects nearly 44 million people and is one of major causes of age-dependent disability [14]. With no definitive cure or treatment course in sight, the global cost of Alzheimer’s and dementia is estimated to be $605 billion. The pathological characteristics of AD present as cortical atrophy, neuro-inflammation, neuronal cell death, loss of synaptic connections, and the accumulation of neurofibrillary tangles and senile plaques [15].

The exact cause of AD is still unknown; however, genetic mutations in APP, presenilin 1 (PS1), or presenilin 2 (PS2) have been shown to promote the formation of amyloid plaques, which are a hallmark of AD pathology [16, 17]. The apolipoprotein E (ApoE) gene encodes three isoforms: ApoE2, ApoE3, and ApoE4. The ApoE4 isoform is identified as a genetic risk factor for late-onset AD because of its impact on the clearance of Aβ and amyloid deposition [18–21]. ApoE can also impact Aβ accumulation through its receptor, as ApoE receptor knockout mice have shown to increase Aß accumulation due to reduced clearance [22].

On the other hand, some AD risk genes are also involved in synaptic plasticity; loss of synaptic function in AD is evident long before any substantial loss of neurons [23]. PS1 is a component in the synaptic junction [24] and has been shown to regulate calcium homeostasis and the release of certain neurotransmitters [25–28]. An AD mouse model with a PS1 mutation also exhibits disruption in homeostatic scaling, a mechanism for preventing groups of neurons from altering their firing patterns too drastically in response to changes in the environment [29]. ApoE may also regulate synaptic functions through its receptor, ApoE receptor 2 (ApoER2), which is known to promote synaptic plasticity and memory formation in mice [30]. Toxic soluble Aß can also be directly cleared from the synapse via ApoE receptors [31]. In mouse models, ApoER2 increases the number of dendritic spines and synapses and stabilizes them by regulating the assembly of a complex of proteins involved in synaptic terminals across neurons, a process which is important for learning and memory [32]. In spite of the association of these proteins with synaptic functions, the effect of these genetic mutations on AD cognitive dysfunction remains to be fully established.

Growing numbers of studies suggest that Aβ is likely to be the early effector molecule in AD cognitive dysfunction [see reviews [4, 33, 34]. Although the precise biochemical mechanisms underlying how variously assembled forms of Aβ cause synaptic dysfunction remain to be determined, biochemical and morphological studies have shown accumulation of Aβ at the synaptic terminals [35, 36]. This local accumulation is likely attributable to the fact that BACE1 initiates the generation of Aβ at the synaptic terminals [37]. Elevated levels of BCE1 have been directly correlated with Aβ-induced pathology in AD brains [38–40]. Increased amyloidogenic processing at the expense of nonamyloidogenic processing promotes Aβ accumulation at synapses in AD.

On the other hand, many scaffolding proteins like mGluR proteins, Shank, Homer, and postsynaptic density 95 (PSD95) are known to form complexes at synaptic terminals, and Aβ accumulation at synaptic terminals leads to disruption of these scaffolding protein interactions, resulting in morphological and physiological alterations such as thinning of the synaptic terminals, alteration in the molecular composition of the PSD, and disruption of synaptic signaling pathways [41–45]. Hence, abnormal accumulation of Aβ is largely considered to be toxic to synaptic functions at multiple levels.

## Effects of BACE1 on synaptic functions

BACE1 is indispensable for the generation of Aβ, as germline deletion of the BACE1 gene abolishes the generation of Aβ [46–48]. BACE1 is therefore a molecule that is directly linked to synaptic functions, at least through its effects on Aβ accumulation in cells and synapses. BACE1 is predominantly expressed in brain and is richly expressed by neurons [37, 49, 50]. Accumulation of BACE1 is observed in normal and dystrophic presynaptic terminals surrounding amyloid plaques in brains of AD mouse models and patients, likely causing a vicious cycle by increasing Aβ production near synapses. Because of this, inhibition of BACE1 is logically viewed to reduce Aβ-mediated synaptic dysfunctions and to be potentially beneficial to AD patients. Hence, BACE1 inhibitors are being developed and tested for treating AD patients [51, 52].

However, whether BACE1 inhibition causes any unwanted effects on synaptic function has also attracted significant attention. This knowledge is critical for understanding the efficacy of BACE1 inhibitors in AD patients. It has been shown that BACE1 is normally expressed in broad brain regions, with rich expression by hippocampal granule cells. BACE1 has been shown to play a critical role in synaptic development and plasticity through cleavage of its various substrates [51, 53, 54]. The effects of BACE1 on synaptic functions are likely to be through multiple mechanisms, as discussed below.

1. BACE1 deficiency alters synaptic plasticity in relation to APP cleavage: Long-term changes in the strength of synaptic transmission are the basis of memory formation. Any correlated activity in the pre- and postsynaptic compartments of a synapse in a repeated pattern either strengthens synaptic connections (long term potentiation; LTP) or conversely weakens them (long term depression; LTD) [55]. High levels of Aβ disturbs the balance of reactive oxygen species (ROS) in synaptic boutons and can interfere with pre- and postsynaptic function, presumably by affecting NMDARs, presynaptic P/Q type Ca^2+^ channels, and/or α7-nAChRs, and thus interrupting subsequent Ca^2+^ signaling and leading to altered synaptic function [56].
  Mice lacking the BACE1 gene show no β-secretase activity and thus have nearly abolished Aβ (Aβ40 and Aβ42) production in the brain compared to wild-type controls. A deletion of the BACE1 gene in mouse models of AD was able to rescue hippocampal-dependent memory deficits resulting from Aβ accumulation [49] and to ameliorate impaired hippocampal cholinergic regulation of neuronal excitability [57]. Alternatively, BACE1 cleavage of APP will also produce a 99-amino acid C-terminal fragment, referred to as βAPPc or APP-C99. This βAPPc has been shown to impair synaptic functions [58]. BACE1 deficiency benefits AD patients likely through reducing this toxic fragment. These findings implicate that BACE1 may be a good therapeutic target for treating AD [59, 60].
  However, recent research progress may suggest otherwise. Since BACE1 has normal physiological functions in synaptic transmission and plasticity in CA1 region of hippocampus, BACE1-null mice displays deficits in both synaptic transmission and plasticity at the hippocampal Schaffer collateral to CA1 synapses [49, 61]. There is a significant increase in the pair pulse ratio (PPF) in BACE1-null mice when compared to wild-type [49]. Because changes in PPF ratio have been attributed to alterations in presynaptic release probability [62], the increased PPF ratio seen in BACE1-null mice may indicate a deficit in presynaptic release [49]. Consistently, BACE1-null mice display altered synaptic plasticity in CA1 and CA3 regions [49, 63]. In addition to presynaptic alterations, changes in PPF ratio can also be attributed to postsynaptic modifications, such as in the subunit composition of AMPA receptors (AMPARs) [64]. Physiological concentrations of Aβ (in pM range) have been shown to facilitate synaptic plasticity [65] and BACE1 deficiency will cause a remarkable reduction in Aβ. Such a loss of physiological levels of Aβ may also lead to synaptic deficits.
2. BACE1 deficiency alters synaptic plasticity in relation to neuregulin-1 cleavage: An alternative possibility is that the synaptic dysfunctions in BACE1-null mice may arise from abnormal processing of substrates other than APP, i.e., neuregulin-1 (Nrg1) [66–68]. Nrg1 has a plethora of functions in the central and peripheral nervous systems, which include regulation of myelination, radial and tangential neuronal migration of glutamatergic and GABAergic neurons, and synaptic plasticity [69]. To exert these functions, Nrg1 is required to be cleaved by membrane-anchored proteases, and BACE1 is one such protease. BACE1 deficiency reduces Nrg1 signaling activity and causes defects in these functions as manifested in BACE1-null mice [70, 71].
  Many behaviors in animals with Nrg1 mutations exhibit a close resemblance to putative characteristics of schizophrenia, such as impaired pre-pulse inhibition, and spontaneous hyperactivity, which can be reversed by clozapine [72]. Nrg1 and its signaling receptor, the ErbB4 receptor, have been identified as leading candidates for schizophrenia susceptibility genes [73]. It has also been shown that Nrg1-ErbB4 signaling enhances excitatory synapse formation on interneurons and inhibitory synapse formation on pyramidal neurons [74, 75]. Specifically, deletion of ErbB4 from fast-spiking interneurons, such as chandelier and basket cells, has been shown to cause relatively subtle but consistent synaptic defects [74]. Deletion of ErbB4 in interneurons increases miniature excitatory postsynaptic current (mEPSC) frequency and amplitude, but increases miniature inhibitory postsynaptic current frequency in pyramidal neurons [75, 76]. In addition, Nrg1 increases both the number and size of PSD-95 puncta, indicating that Nrg1 stimulates the formation of new synapses and strengthens existing synapses. Nrg1 could also stimulate the stability of PSD-95 in a manner that requires tyrosine kinase activity of ErbB4 [77]. Together, these results suggest that Nrg1 plays a significant role in excitatory synapse development, possibly via stabilizing PSD-95 [76]. By abolishing Nrg1 cleavage to reduce Nrg1-ErbB4 signaling in synapses, BACE1 deficiency likely contributes to synaptic dysfunctions as reported in BACE1-null mice discussed above.
3. BACE1 deficiency alters synaptic plasticity in relation to Sez6 cleavage: Another family of proteins, the seizure-related gene 6 (Sez6) and its family member Sez6L, were identified as BACE1 substrates through an unbiased proteomic approach and were recently validated as strong substrates of BACE1 [78]. Sez6 and Sez6-like (Sez6L) are nearly exclusively cleaved by BACE1 and not by other proteases in the brain and are guided by their sub-cellular location and their function. They share an NPxY motif and a phosphotyrosine-binding domain (PTB) with another BACE1 substrate, amyloid precursor protein (APP). In BACE1-null and BACE1/2-double-null mice, a marked reduction in the shedding of Sez6 and Sez6L proteins has been confirmed. Their levels in BACE1-null cerebrospinal fluid (CSF) are significantly reduced to ~10% of the wild-type condition. Although the exact molecular functions of Sez6 and Sez6L are not yet fully understood, homology in their protein-binding domains to other cell surface receptors suggests that they may act as receptors at the cell surface [79], as they were originally identified as membrane proteins with five copies of short consensus repeat with a complement C3b/C4b binding site and were seen to be elevated after bursts of neuronal activity [80]. The interaction domains suggest adhesive and/or receptor trafficking functions of these proteins; however, their binding partners are not yet known. Sez-6 is required for normal dendritic arborization of cortical neurons, which is critical for neuronal transfer of information. Its localization along developing and mature dendritic branches and in dendritic spines modulates branch stability. In the absence of Sez-6, mice exhibit short dendrites while cultured cortical neurons display excessive neurite branching. Despite the noticeable effect on branching of dendrites, no obvious effect on an overall growth of the dendritic arbor is reported [81]. Excessive dendritic branching does not always mean a better condition for synapse formation, as studies have found that postsynaptic specializations on these branches (labeled with PSD-95) were dramatically reduced [82]. In the absence of Sez-6, spine numbers are reduced, with reduced excitatory synaptic connectivity between layers II/III and layer V pyramidal neurons in Sez-6-null mice. As spontaneous miniature EPSCs (mEPSCs) or EPSCs with minimal stimulation were not altered, the reduction might be because of uncoupling of pre- and postsynaptic ends of synapses due to altered branching patterns. There is also evidence for reduced synaptic density, punctate staining of PSD-95, and LTP in the frontal cortex of Sez6-null mice. Sez-6 proteins are therefore important for specifying proper dendritic arborization and for development of excitatory synapses on cortical neurons [81]. There is an activity-driven up-regulation of Sez-6 expression after 2 h post-high frequency stimulation [83]. Sez-6 expression levels are highly enriched in brain regions associated with ongoing morphological plasticity, such as the hippocampus and cerebellum in postnatal brain. In Sez-6 deficiency, animals exhibit poor motor coordination and balance, suppressed activity in the open field, reduced anxiety, as well as cognitive deficits. Thus Sez6 protein signaling is critical for excitatory synapse development and function [81] and synaptic circuit refinement [84]. Besides synapse formation and maintenance, Sez6 family members are also expressed and cleaved in lungs and pancreas [79, 85]. Since Sez6 and Sez6L are exclusive substrates of BACE1, they can be used as a direct readout for BACE1 activity in CSF and as a control condition where BACE1 inhibitors can be developed in a substrate-specific manner (for APP) without hampering the physiological actions of BACE1 on other essential proteins like Sez6 that are critical for proper synchronous synaptic transmission.
4. BACE1 deficiency alters synaptic plasticity in relation to jagged cleavage: Jagged-1 (Jag1) has been identified as a BACE1 substrate [86] and is known to play important roles in neural development and synaptic functions. Jag1 regulates astrogenesis/neurogenesis via the Notch signaling pathway [87–89]. Because of abrogated Jag1 cleavage, BACE1-null mice exhibit increased astrogenesis and reduced neurogenesis due to increased Jag1-Notch interactions [87]. This is consistent with a prior report that astrocytes negatively regulate neurogenesis through the Notch pathway [90]. Although Notch and its ligands are expressed at low levels in the adult brain [91, 92], they are needed for long-term memory, which is dependent on ultra-structural remodeling of synapses. Hence Notch has an important role in the neural plasticity underlying consolidated memory. Loss of Notch function produces memory deficits in *Drosophila melanogaster* [93] and impairs proper morphology of dendritic spines [91] in the mouse hippocampus. Thus Jag, as a Notch regulator, is important for synaptic plasticity that contributes to memory formation.
  On the other hand, a shift in the balance between neurogenesis and astrogenesis in BACE1-null mice likely contributes to aberrant synaptic transmission. Astrocytes regulate synaptic function and plasticity in close association with synapses [94]. They are involved in synaptogenesis as well as synapse function and elimination. This tight structural and functional partnership between the perisynaptic astrocytic process and the neuronal pre- and postsynaptic structures constitutes the “tripartite synapse” [95]. Astrocyte processes enclose synapses and define functional domains by ensheathing neuronal somas, axons, dendrites, and synapses occupying non overlapping territories, and thus establish gradually independent domains which are also developmentally regulated [96, 97]. This process of segregation, also known as astrocyte tiling, is thought to be regulated by “contact inhibition” between neighboring astrocytes and is crucial for normal functions of the nervous system because, in disease and post-injury conditions, astrocytes lose their tiling ability and display intermingled process morphology [98]. Astrocytes have also been known to regulate glutamatergic postsynaptic strength by increasing the number and stabilizing of AMPAR and NMDAR at the postsynaptic end of synapses [99]. Hence, BACE1 inhibition may impact synaptic functions due to an imbalance in total astrocytes and neurons.

## Conclusion

Since BACE1 is the rate-limiting enzyme in the amyloid cascade, it is considered to be one of the promising targets for AD therapy. A rare human mutation at the BACE1 cleavage site of APP has been identified, which results in a 40% decrease in Aβ production in vitro, a reduced propensity of Aβ to aggregate, a five- to seven-fold reduced risk of developing AD, and improved cognitive function in elderly subjects without AD [100–102]. Hence, BACE1 inhibition is likely to be beneficial to AD patients. However, caution should also be taken considering the role of BACE1 in synaptic plasticity. For example, the BACE1 inhibitor verubecestat (MK-8931) showed great promise in early human and animal trials [103], but a recent announcement that Merck was stopping one of its trials suggested cause for concern. By better understanding the physiological and pathological functions of BACE1, anticipation and possible circumvention of mechanism-based side effects that may arise due to BACE1 inhibition can be accomplished. Decoding molecular mechanisms that underlie AD pathogenesis will help us to develop efficient therapeutic approaches to combat disease progression.
